# Prevalence of bacterial vaginosis and its associated risk factors among women of reproductive age attending Jos University Teaching Hospital, Plateau State, Nigeria

**DOI:** 10.3205/dgkh000580

**Published:** 2025-09-22

**Authors:** Florence Yachim Danjuma, Michael Macvren Dashen, Anayochukwu Chibuike Ngene, Otumala John Egbere

**Affiliations:** 1 Department of Microbiology, Faculty of Natural Sciences, University of Jos, Jos, Nigeria; 2 Department of Microbiology, College of Natural Sciences, Michael Okpara University of Agriculture, Umudike, Nigeria

**Keywords:** bacterial vaginosis, vaginal flora, reproductive-aged women, prevalence, risk factors, Nugent scoring system

## Abstract

**Introduction:**

Bacterial vaginosis (BV) remains the most common cause of abnormal vaginal discharge due to altered vaginal flora with decreased *Lactobacillus* spp. and increased anaerobic bacteria. The objectives of this study were to establish the prevalence of BV and its risk factors among women of reproductive age.

**Method:**

This cross-sectional descriptive survey was conducted among 220 non-pregnant women of reproductive age attending Jos University Teaching Hospital (JUTH) in Plateau state, north central Nigeria, between August 2021 and January 2022, in which 110 women were asymptomatic, and 110 women were symptomatic. Self-administered questionnaires were used to identify the sociodemographic status and predisposing factors of the participants. Positive BV diagnosis was made using the Nugent scoring system and bacterial species were identified on selective media. A descriptive analysis was performed using the Chi-squared test at a 95% confidence interval to determine the prevalence of BV and its associated risk factors.

**Results:**

The overall BV prevalence was 33.6%. BV was higher in symptomatic women (39.1%) than in asymptomatic women (28.2%). No correlation was found between BV and demography, knowledge, or health behaviour, including age, education, marital status, number of children, occupation, income, alcohol, tobacco smoking, or sexual as well as hygienic behaviour (p≥0.05).

A total of 328 bacterial isolates from 16 species were identified, with *Enterococcus (E.) faecalis* being the most prevalent species, accounting for 39.3% of the total isolates. Other species isolated include *Staphylococcus* *(S.) saprophyticus* (13.6%), *S. epidermidis* (13.0%), *S. aureus* (4.7%), *Streptococcus (Sr.) agalactiae* (5.9%), *Klebsiella (K.) pneumoniae* (4.1%), *Proteus (P.) mirabilis* (2.4%), and *Pseudomonas (P.) aeruginosa* (3.6%) in the symptomatic group. *S. saprophyticus* (10,7%), *S. aureus* (3.8%), *K. pneumoniae* (2.5%), and *P. aeruginosa* (1.9%) were isolated in the asymptomatic group. *P. mirabilis* was not detected. *Lactobacillus* spp. were present but not dominant, with an overall prevalence of 8.2%.

**Conclusion:**

The observed diversity in vaginal microbiota, particularly the higher prevalence of *E. faecalis* in asymptomatic women, suggests the complexity of microbial interactions. The low prevalence of *Lactobacillus* spp. indicates a potential risk for infections, while the presence of potentially pathogenic bacteria such as *S. saprophyticus* and *Streptococcus agalactiae* underscores the need for further research. Overall, the understanding of the vaginal microbiome is crucial for developing effective healthcare interventions for managing BV.

## Zusammenfassung

**Einleitung:**

Bakterielle Vaginose (BV) ist nach wie vor die häufigste Ursache für anormalen Vaginalausfluss, der auf eine veränderte Vaginalflora mit Abnahme der Lactobacillus Arten und Zunahme anaerober Bakterien zurückzuführen ist. Ziel der Studie war es, die Prävalenz von BV und deren Risikofaktoren bei Frauen im reproduktiven Alter zu ermitteln.

**Methode:**

Es handelt sich um eine deskriptive Querschnittserhebung unter 220 nicht schwangeren Frauen im reproduktiven Alter, die zwischen August 2021 und Januar 2022 das Jos University Teaching Hospital (JUTH) im Bundesstaat Plateau in Nord-Zentral-Nigeria besuchten. 110 Frauen waren asymptomatisch und 110 Frauen waren symptomatisch.

Anhand von selbst ausgefüllten Fragebögen wurden der soziodemografische Status und prädisponierende Faktoren der Teilnehmerinnen ermittelt. Eine positive BV-Diagnose wurde anhand des Nugent-Scoring-Systems gestellt. Die Bakterienarten wurden auf Selektivmedien identifiziert. Zur Bestimmung der Prävalenz von BV und der damit verbundenen Risikofaktoren wurde eine deskriptive Analyse mittels Chi-Quadrat mit 95% Konfidenzintervall durchgeführt.

**Ergebnisse:**

Die Prävalenz der BV betrug 33,6%. Die BV war bei symptomatischen Frauen (39,29%) häufiger als bei asymptomatischen Frauen (28,2%). Es war keine Korrelation zwischen BV und Demographie, Wissen, Gesundheitsverhalten einschließlich Alter, Bildung, Zivilstand, Parität, Beruf, Einkommen, Alkohol, Tabakkonsum und Sexual-sowie Hygieneverhalten nachweisbar (p≥0,05).

Insgesamt wurden aus 328 Bakterienisolaten 16 Bakterienarten identifiziert. Dabei war *Enterococcus (E.) faecalis* mit 39,3% der Gesamtzahl der Isolate die häufigste Art. Der Anteil von St*aphylococcus (S.) saprophyticus* betrug 13,6%, von *S. epidermidis* 13,0%, *S. aureus* 4,7%, *Streptococcus (Sr.) agalactiae* (5.9%), *Klebsiella (K.) pneumoniae* 4,1%, *Proteus (P.) mirabilis* 2,4% und *Pseudomonas (P.) aeruginosa* 3,6% in der symptomatischen Gruppe. In der asymptomatischen Gruppe betrug der Anteil von *S. epidermidis* 11,9%, *S. aureus* 3,8%, *K. pneumoniae* (2.5%) und *P. aeruginosa* 1.9%. *P. mirabilis* wurde nicht nachgewiesen. Lactobacillus Arten waren mit einer Gesamtprävalenz von 8,3% präsent, aber nicht dominant.

**Schlussfolgerungen:**

Die beobachtete Vielfalt der vaginalen Mikrobiota, insbesondere die höhere Prävalenz von *E. faecalis* bei asymptomatischen Frauen, verdeutlicht die Komplexität der mikrobiellen Interaktionen. Die geringe Prävalenz von Lactobacillus spp. deutet auf ein potenzielles Infektionsrisiko hin, während das Vorhandensein potenziell pathogener Bakterien wie *S. saprophyticus* und *Streptococcus agalactiae* den Bedarf an weiteren Untersuchungen unterstreicht. Insgesamt ist das Verständnis des vaginalen Mikrobioms von entscheidender Bedeutung für die Entwicklung wirksamer Gesundheitsmaßnahmen zur Behandlung von BV.

## Introduction

Bacterial vaginosis (BV) is often referred to as a polymicrobial syndrome, most predominantly characterized by a decline in lactobacilli count and a rise in the density and variability of obligatory/facultative anaerobes, including *Gardnerella* spp., *Atopobium* spp., *Prevotella* spp., and *Mobiluncus* spp. [1], [2], [3]. BV presents serious clinical hazards for female reproductive health. In most cases, BV may produce no symptoms, but this condition can have several consequences and raise certain health issues. BV also makes women more vulnerable to a variety of gynecological and obstetric complications, such as infections [4] infertility [5] and premature birth [6].

BV is the most common vaginal infection; it affects more than one-third of women of child-bearing age [7], [8]. It also implies that the incidence of BV may differ with different population groups and geographical locations. Cross-sectional epidemiologic surveys have documented BV prevalence ranging from as low as 10% to as high as 50% [9], [10]. BV prevalence tends to be higher in parts of Africa and the lower in many regions of Asia and Europe. Nonetheless, some African populations report low prevalence rates, while certain Asian and European populations report relatively high rates [11]. In the United States, the estimated prevalence among 21.2 million women aged 14–49 years was 29.2%, based on data from a nationally representative sample in the NHANES 2001–2004 survey [12].

BV may be precipitated by behaviours such as sexual intercourse, hormonal fluctuations, and cleanliness. Women who are sexually active, particularly those with multiple sexual partners, have a higher risk of the occurrence of BV [13], [14]. Some studies have shown that vaginal douching seems to increase the incidence of BV [15], [16]. Research on ethnicity reveals that African American and Hispanic women are more prone to BV than are Caucasian women [17]. These disparities could be attributed to genetic, cultural, and even socioeconomic factors [18]. Smoking, low economic status, hormonal imbalances and the presence of previous sexually transmitted infections (STI) are also believed to be causes of BV [19], [20].

Because BV is a prevalent disease worldwide and can cause several complications, it is a major concern for global public health. The global burden of BV can be observed through the effects it has on women’s sexual and reproductive health mostly among low- or middle-income countries. It is uncommon in developed countries. In sub-Saharan Africa, the burden of BV is compounded by HIV, for which BV is a cofactor in increasing the risk of HIV infection and transmission [21]. Prior works have reported a high prevalence of BV in Nigeria; pregnant women are especially at risk of experiencing adverse pregnancy outcomes, such as preterm birth and low birth weight [22], [23]. The high incidence of BV in Nigeria can be ascribed to the following factors: poor hygiene, little or no access to appropriate health facilities, and unsafe sex [24].

Despite being one of the most common vaginal infections globally, BV remains under-diagnosed and undertreated. BV affects approximately 20–30% of women of reproductive age worldwide, with prevalence rates as high as 50% in some regions. Despite treatment, up to 50% of women experience recurrence within six months, leading to ongoing health complications and reduced quality of life [25]. The high prevalence, recurrence, and association with serious health complications make BV a significant public health issue that warrants further investigation. Therefore, investigating the burden of BV is crucial to address the public health challenges associated with this condition. 

Thus, the aim of this study was to determine the prevalence of BV and its associated risk factors among women of reproductive age attending a teaching hospital in Plateau State, Nigeria. Understanding the prevalence of BV and its associated risk factors among reproductive-age women is crucial for developing effective prevention strategies, appropriate diagnostic measures, and targeted interventions. 

## Methods

### Study design

The descriptive cross-sectional study was conducted between August 2021 and January 2022 among 220 women of childbearing age visiting Jos University Teaching Hospital, Plateau State. 

### Ethical approval

The study received ethical approval from the JUTH ethics committee (Ref. JUTH/DCS/IREC/127/XXX/2478). In addition, oral informed consent was obtained from the participants before enrollment. All the procedures conducted complied with the ethical standards of the Federal Ministry of Health in Nigeria. 

### Selection criteria 

The study involved women of childbearing age – 18–50 years – attending for annual cervical cancer screening, or women with complaints of conditions that might include vaginal infections, if they agreed to participate. Women who were younger than 18 or older than 50 years, women who were menstruating or pregnant, and those who were receiving antibiotics or antifungal treatment at the time of sample collection were not included in the study. 

### Questionnaire

Sociodemographic data and predisposing factors were obtained from the participants using structured questionnaires. Those who could not read and write were assisted by face-to-face interviews to fill in the questionnaire.

### Sample collection and processing

A total of 220 vaginal swabs were obtained from the participating women completing the questionnaires and meeting enrolment criteria. From 110 women presenting with complaints of vaginal infections, 110 swabs were sampled. Furthermore, from 110 women lacking such complaints and undergoing routine cervical smears, 110 swabs were also collected. The lateral vaginal walls were sampled with sterile cotton tips to obtain cultures. Each sample was collected in duplicates: One swab was utilized to undergo Gram staining, followed by microscopy, while the second swab was dipped in 2 ml of Amies gel transport medium and then transported in ice packs to the laboratory for bacterial culture. 

### Microscopic examination of vaginal swabs for BV

The presence of BV was determined using the Nugent scoring criteria. The vaginal swab sticks were smeared on microscopic slides, air-dried, heat-fixed, and Gram-stained. The Gram-stained slides were then examined under an oil-immersion objective (1,000x magnification) and graded as per the standardized, quantitative, morphological classification developed by Nugent. Composite scores were grouped into three categories: scores 0–3: negative for BV; 4–6: indeterminate for BV; 7–10: indicative of BV [26] (Table 1 (Tab. 1) and Table 2 (Tab. 2)).

### Isolation of bacteria species

Isolation was done following procedures described previously by Edet et al. [2] and Ranjitet al. [15]. From the 2-ml transport-medium suspension containing the vaginal swab, a sterile cotton swab stick was dipped into the medium and inoculated unto plates containing freshly prepared de Man-Rogosa Sharpe agar (MRS agar), blood agar, and chocolate agar. The inoculated plates were placed in an anaerobic jar and then incubated at 37°C for 24 hours. Similarly, a swab of the suspension was inoculated onto plates containing freshly prepared eosin-methylene blue agar and mannitol salt agar, then incubated at 37°C for 24 hours under aerobic conditions. After incubation, colonies were randomly selected based on their morphology on each plate and sub-cultured, then Gram stained and finally stored for further biochemical characterization. 

### Statistical analysis

A descriptive analysis was performed using the Chi-squared test at a 95% confidence interval (CI) to determine the prevalence of BV and its associated risk factors. The analysis was conducted using SPSS software version 25.

## Results

### Prevalence of BV and sociodemographic characteristics

The distribution of study participants’ sociodemographic characteristics is shown in Table 3 (Tab. 3), Table 4 (Tab. 4), Table 5 (Tab. 5), Table 6 (Tab. 6), Table 7 (Tab. 7), and Table 8 (Tab. 8). Altogether, 110 non-pregnant women who were asymptomatic and 110 women who were symptomatic took part in the study. BV was diagnosed in 28.2% of asymptomatic women and in 39.1% of the symptomatic ones. 

Among the asymptomatic group, the highest BV prevalence (53.4%) was observed in the 46–50 age group. In contrast, the symptomatic group recorded the highest prevalence (48.6%) in the 36–45 age group. However, the p-values for the asymptomatic and symptomatic groups were 0.133 and 0.535, respectively, indicating that the association between age group and BV prevalence was not statistically significant (p>0.05).

With respect to education level, the highest BV prevalence in the asymptomatic group was observed among non-respondents (66.7%), while in the symptomatic group, those with only primary education had the highest prevalence (55.6%). However, there was no statistically significant association between education level and BV prevalence (p>0.05).

There was no relationship between marital status and BV prevalence in the two groups. The BV-positive rate was highest among the widowed (50.0%) in the asymptomatic group, and the divorced (50.0%) or separated (50.0%) in the symptomatic group. Likewise, the number of children, employment status and income level did not show any statistically significant relationship with BV prevalence (p≥0.05).

### Prevalence of BV and life style

The results for the relationship between lifestyle factors and BV are presented in Table 9 (Tab. 9). Among asymptomatic women, there were no cases of BV among those who reported regular alcohol consumption, 15.4% of those who occasionally consumed alcohol tested positive for BV, while among those who never consumed alcohol, the prevalence was 29.9%. The p-value for the association between alcohol consumption and BV prevalence in asymptomatic women was 0.275.

In the symptomatic group, 1 out of the 2 women who reported regular alcohol consumption tested positive for BV, yielding a prevalence of 50.0%, while among those who consumed alcohol occasionally, the prevalence was 20.0%. Among women who never consumed alcohol, 41.9% were BV-positive. The p-value for the association between alcohol consumption and the prevalence of BV in symptomatic women was 0.258.

Among asymptomatic women, there were no cases of BV observed in any of the smoking categories, so that no p-value was recorded. All the women reported not smoking at all. In the symptomatic group, 1 out of the 2 women who reported smoking occasionally tested positive for BV, while there were no participants who reported smoking regularly, hence, no case was recorded. Among women who never smoked, 38.9% were positive for BV. The p-value for the association between smoking status and BV prevalence in the symptomatic group was 0.750.

### Prevalence of BV and hygiene practices

Among asymptomatic women, 5.5% reported washing their vagina once daily, 24.6% reported washing 2 times daily, 12.7% reported washing 3 times daily and 51.8% reported washing more than 3 times daily. The prevalence rates for women who washed their vagina once daily, 2 times daily, 3 times daily and more than 3 times daily were 33.3%, 2.2%, 35.7% and 28.1% respectively. The p-value for the association between the frequency of vaginal washing and BV prevalence in the asymptomatic group was 0.906 (Table 10 (Tab. 10)).

In the symptomatic group, 6.4% reported washing their vagina once daily, 26.4% reported washing 2 times daily, 10.9% reported washing 3 times daily and 53.6% reported washing more than 3 times daily. The prevalence rates for women who washed their vagina once daily, 2 times daily, 3 times daily and more than 3 times daily were 14.3%, 48.3%, 33.3% and 39.0% respectively. The p-value for the association between the frequency of vaginal washing and prevalence of BV in the symptomatic group was 0.550.

The correlation between the prevalence of BV and use of antiseptics is presented in Table 11 (Tab. 11). Among asymptomatic women, 4.5% reported regular use of antiseptics, 15.5% reported occasional use and 74.6% reported they never use antiseptics. The BV positivity rates for women who regularly and occasionally used antiseptics were 33.3% and 29.4%, respectively. The p-value for the association between antiseptic use and BV prevalence in the asymptomatic group was 0.917. In the symptomatic group, 6.4% of women reported regular use of antiseptics and 26.4% reported occasional use. The prevalence rates for women who regularly and occasionally used antiseptics were 28.6% and 51.7%, respectively. The p-value for the association between antiseptic use and BV prevalence in the symptomatic group was 0.917.

Table 12 (Tab. 12) and Table 13 (Tab. 13) show the BV prevalence by specific products used for vaginal washing and use of vaginal deodorant, respectively. In the asymptomatic group, 10.9% of women reported using regular hand soap for vaginal washing; BV prevalence in this subgroup was 16.7%. The use of medicated soap was reported in 3.6%, and 25.0% of these participants were BV positive. Also, 5.5% of women reported using vaginal products for washing, but none of them tested positive for BV. In the symptomatic group, 12.7% reported using regular hand soap, and 35.7% of these were positive for BV. 9.1% reported using medicated soap, with a BV prevalence rate of 30.0%; 14.6% reported using vaginal products, and 62.5% of these participants were BV positive.

Most women in both the asymptomatic (72.7%) and symptomatic (60.0%) groups reported using water only for vaginal washing. Among asymptomatic women using water only, 31.3% tested positive for BV, while in the symptomatic group, the BV-positive rate was 36.46%. The p-value for the association between the products used for vaginal washing and BV prevalence in both asymptomatic and symptomatic group were 0.561 and 0.327, respectively.

Among asymptomatic women, 86.4% reported never using vaginal deodorant, while 7.3% reported occasional use and 1.8% reported regular use. The BV positivity rates for regular and occasional users were 50.0% and 25.0% respectively. The p-value associated with the use of deodorant and BV prevalence in the asymptomatic group was 0.829. In the symptomatic group, 2.7% of women reported regular use of deodorant, while 6.5% reported occasional use. The BV positivity rates for regular and occasional users were 33.3% and 28.6%, respectively. The p-value for the association between deodorant use and BV prevalence in the symptomatic group was 0.8535.

### Prevalence of BV and sexual activities

In the asymptomatic group, the highest BV prevalence (37.5%) was seen among those with two sexual partners, while the lowest (25%) was in those with more than two partners. In the symptomatic group, two sexual partners were linked to the highest prevalence (50%), followed by one partner (42.5%). There was no significant association between sexual partners and BV prevalence in either group (asymptomatic: p=0.942; symptomatic: p=0.164).

Among asymptomatic participants, those who used lubricant occasionally had the highest BV prevalence (41.7%), with no cases in regular users. In the symptomatic group, occasional lubricant use also showed the highest prevalence (50%). Chi-square tests showed no significant association between lubricant use and BV in either group (asymptomatic: p=0.645; symptomatic: p=0.696).

Contraceptive use did not show a significant difference in BV prevalence in either group. In the asymptomatic group, 29.6% of non-users and 26.8% of users tested positive. In the symptomatic group, 40.3% of non-users and 36.8% of users tested positive (asymptomatic: p=0.759; symptomatic: p=0.252).

For oral sex, regular practitioners in the asymptomatic group had the highest BV prevalence (50%), while the lowest was in those who never practiced it (28.8%). In the symptomatic group, regular practitioners had the lowest prevalence (25%), with the highest seen in non-practitioners (43.8%). No significant association was found between oral sex and BV in either group (asymptomatic: p=0.880; symptomatic: p=0.245) (Table 14 (Tab. 14)).

### Occurrence of bacterial species

A total of 328 organisms were isolated from the study (159 from asymptomatic women and 169 from symptomatic women). Among the isolates, *E. faecalis* appeared to be the most prevalent bacterial species in both groups, accounting for 39.3% of the total isolates (Table 15 (Tab. 15)). The organism was more prevalent in the asymptomatic group (45.3%) compared to the symptomatic group (37.7%). This was followed by *S. epidermidis* with 12.5% and* S. saprophyticus* with 12.2%. *S. epidermidis* was more frequently isolated from symptomatic women (13.0%) compared to asymptomatic women (11.9%). Likewise, *S. saprophyticus* was more frequently isolated from symptomatic women (13.6%) compared to asymptomatic women (10.7%).

There was a higher prevalence of potentially pathogenic bacteria – such as *S. aureus* (4.7%), *K. pneumoniae* (4.1%), *P. mirabilis* (2.4%), and *P. aeruginosa* (3.6%) – in the symptomatic group compared to the asymptomatic group, with *S. aureus*, *K. pneumoniae*, *P. mirabilis*, and *P. aeruginosa* frequencies of 3.8%, 2.5%, 0%, and 1.9%, respectively (Table 15 (Tab. 15)).

In the asymptomatic group, *Lactobacillus* (l) spp. were present but not dominant. The most common species included *L. plantarum* (3.1%), *L. fermentum* (2.5%), and *L. curvatus* (1.3%). There was a lower presence of *Lactobacillus* spp. in the symptomatic group. *L. plantarum* (2.4%) and* L. rhamnosus* (1.8%) were present, but at lower frequencies compared to the asymptomatic group. Overall, *Lactobacillus* spp. had a total prevalence of 8.2%, with a higher prevalence in the asymptomatic group (9.4%) as compared to symptomatic group (7.1%) (Table 15 (Tab. 15)). *P. mirabilis* showed a significant difference between asymptomatic and symptomatic groups (p=0.045). All other organisms had p-values greater than 0.05, indicating no significant differences between the groups for those organisms.

## Discussion

### Prevalence

A total of 220 non-pregnant women were recruited, half of whom had no symptoms of cervicovaginal inflammation (n=110), while the other half did (n=110). The percentage estimate of total BV at the population level was 33.6%, with 39.1% in the symptomatic group and 28.2% in the asymptomatic group. The results align with existing literature, which reports that BV is more commonly observed in symptomatic women than in asymptomatic ones [10], [13], [27]. This higher prevalence among symptomatic women may be attributed to the clinical symptoms (such as vaginal discharge, odour, and irritation) that are characteristic of BV, which likely prompt women to seek medical attention, leading to a higher diagnosis rate. The prevalence of BV in this study was higher (33.6%) compared to a study conducted in Ethiopia, which found a lower prevalence of 19.4% [12]. Similarly, a study in sub-Saharan Africa showed a lower prevalence of BV among a well-defined group of women [28]. In that study, which included 1,404 women from South Africa, Rwanda, and Tanzania, the overall BV rate was found to be 23.2%. The prevalence of BV varied across the countries in that study, with the highest prevalence in South Africa (29.7%), followed by Rwanda (21.9%), and Tanzania (19.3%) [28]. In contrast, other sub-Saharan countries reported higher BV prevalence rates, such as Kenya (37%) [29], Botswana (38%) [30], and Zimbabwe (35.5%) [31]. To determine the severity of the condition, it is relevant to identify both the rates of BV in women with symptoms as well as in those who are asymptomatic. Next, having proved that BV is present in the study population, it is important to attempt to determine the relationship between BV and factors that may influence BV rate. 

### Sociodemographic characteristics

In this study, the highest prevalence of BV in the asymptomatic and symptomatic groups was observed in the 46–50 and 36–45 age groups, respectively, with prevalence of 53.45% and 48.6%. These findings are consistent with previous research that indicates higher BV prevalence in these age groups [15], [32], potentially due to factors such as hormonal changes, sexual activity, and age-related alterations in vaginal flora [33], [34]. However, this study found no statistically significant relationship between age and BV prevalence (p≥0.05), supporting the findings of Bahram et al. [35], who also concluded that age does not significantly correlate with BV. This suggests that age may not be a strong independent risk factor for BV in this cohort, and the observed variations in prevalence across different age groups could be incidental. Other studies have also investigated the association between age and bacterial vaginosis, and the results have been inconsistent. While some studies have reported a significant association with age [28], [36], [37], others have not found a significant association [10], [38]. These discrepancies may be attributed to variations in study populations, geographic locations and methodological differences.

According to the results obtained in this study, educational level did not correlate with BV prevalence in either asymptomatic or symptomatic women. There was no statistically significant association in the symptomatic group, although the group with the highest prevalence (55.6%) had only attained a primary-school education. The lack of association means that having a higher educational level it is not sufficient to determine the prevalence of BV. These findings are in line with a different study, which observed that educational level does not explain BV prevalence [38]. However, Koumans et al. [37] noted a significant link between educational level and BV. Women with lower educational attainment (less than a high school education) had higher rates of BV. Further analysis of other demographic factors, including marital status, number of children, employment status, and income level, revealed no significant relationships with BV in this study. The lack of significant associations with these sociodemographic factors suggests that BV is a polymicrobial infection influenced by a variety of agents and is unlikely to be attributed solely to sociodemographic characteristics.

### Lifestyle

The results indicate that there is no association between BV and lifestyle factors (alcohol consumption and tobacco smoking) among either asymptomatic or symptomatic women. This implies that these specific lifestyle practices are not strongly linked with BV or its persistence. The observations are consistent with prevailing literature on the lack of a relationship between alcohol consumption and BV [15], [39], [40]. In the same regard, the study by Ranjit et al. [15] also concluded that no correlation existed between tobacco smoking and BV.

These results shed light on the necessary points in assessment of risk factors and pathogenesis of BV. Although there are multiple other health effects of alcohol consumption and tobacco smoking, they have no influence on the prevalence of BV. It also emphasizes that BV is multifactorial disorder which interacts with several other parameters, e.g., genetic, environmental and microbial [9], [12].

### Sexual activities

In the analysed cohort, the number of sexual partners, the use of vaginal lubricant, use of contraceptives or practice of oral sex do not seem to favour the development of BV or the manifestation of symptoms thereof. Earlier, similar studies indicated that specific sexual behaviours are associated with the disease, but in this survey, certain evidence is lacking. For instance, prior research established that women with many sexual partners tend to develop BV than do women with a single partner [13], [41]. Furthermore, Bahram et al. [35] found an increased risk of BV with the use of contraceptives. However, in this study, no relationship was established between the above factors and BV prevalence. Some previous works have also reported the occurrence of BV among women who are sexually inactive or even virgins [42], [43].

Likewise, although several past investigations have found that a potential relationship may exist between the utilization of vaginal lubricants and BV, the present study did not, which agrees with other authors reporting no such connection [14], [44]. It also shows here that as long as vaginal lubricants are employed in a proper manner and have produced no negative side effects, they do not seem to predispose any woman to BV.

It is, however, important to point out that sexual activities and the usage of vaginal lubricants do result in the shift of vaginal microbiota within a short time and increase the risk of BV, according to some research [12], [15], [35]. Nevertheless, the current study did not record any association; thus, other factors may be more influential in the development of BV in this population.

### Hygiene practices

Regarding the frequency of vaginal washing, there was no association between the number of times women washed their vagina daily and the prevalence of BV. This finding is consistent in both asymptomatic and symptomatic groups. Like our findings, Lehtoranta et al. [19] reported no significant association between the frequency of vaginal washing and BV prevalence. However, they observed a significant association between the use of intimate wash products and an increased risk of BV. This contrasts with our study, which did not find any association between specific products for vaginal washing and the prevalence of BV.

Similarly, our study, along with that by Trabert and Misra [44], found no association between the use of antiseptics and BV prevalence. However, Brotman et al. [14] found that the use of antiseptics was associated with an increased risk of BV. 

The study also examined the specific products used for vaginal washing, including regular hand soap, medicated soap, and vaginal products. No associations were found between the use of these products and the prevalence of BV. This finding is consistent with other studies which also reported no significant associations [45], [46], [47]. However, it is worth noting that some authors have reported conflicting results. For instance, Brotman et al. [14] found that the use of certain products, such as medicated soap, was associated with an increased risk of BV.

Regarding the use of vaginal deodorants, whether women regularly, occasionally, or never used vaginal deodorant, there were no significant differences in BV prevalence rates. Previous studies are contradictory regarding the association between vaginal hygiene practices and BV. Some studies showed a potential link between certain practices and an increased risk of BV [14], [48], [49], while others, including the current study, found no associations [50], [51]. Further research is needed to better understand the complex interactions between vaginal hygiene practices, the vaginal microbiota, and BV.

### Prevalence of bacterial species isolated

This study highlighted the diversity of isolates and potential differences between symptomatic women with vaginal infections and asymptomatic women attending routine visits for cervical cancer screening.

One of our key findings was that *E. faecalis* was the most prevalent species, accounting for 39.3% of the total bacterial isolates. Surprisingly, *E. faecalis* was more frequently isolated from asymptomatic women (72 isolates) compared to symptomatic women (57 isolates). The presence and role of *E. faecalis* in the vaginal microbiota have been explored in several studies. For instance, Ravel et al. [52] analysed the vaginal microbiomes of a large cohort of women and found that enterococci were present in a significant proportion of the vaginal ecosystem. They reported a vaginal community dominated by *Enterococcus* spp., including *E. faecalis*. Similarly, Alioua et al. [53] examined the vaginal microbiota of pregnant women and identified *E. faecalis* as part of the core community of bacteria in the vagina. Other studies also observed an increase in *Enterococcus* spp., as well as other microorganisms, such as *Staphylococcus* spp. and *Sr. agalactiae*, in the vaginal secretions of healthy women [54], [55].

*E. faecalis* plays a crucial role in maintaining the homeostasis of the gastrointestinal (GI) tract by regulating intestinal pH, producing vitamins, and metabolizing nutrients such as carbohydrates, lipids, proteins, and sugars. Additionally, it contributes to the elimination of pathogenic bacteria within the intestines, thereby protecting the human body from various infections and inflammatory responses. While the GI tract is the primary habitat for *E. faecalis*, it is also considered a commensal organism in other parts of the human body, including the genitourinary tract, particularly the vaginal tract [56]. Nonetheless, *E. faecalis* is a frequent colonizer of the vagina; therefore, it is possible for this bacterium to be pathogenic under some circumstances. The concept of commensal pathogens actually becoming pathogenic as a result of specific immune suppression or disturbances in the vaginal flora, or due to specific virulence factors of certain strains, becomes relevant here [57].

*S. saprophyticus* and *S. epidermidis* were the next most prevalent bacterial species, accounting for 12.2% and 12.5% of the isolates respectively. The prevalence rate for *S. epidermidis* was slightly higher in the symptomatic (13.0%) than in the asymptomatic group of women (11.9%). *S epidermidis* is a part of the resident skin and mucous membrane flora. But it can become pathogenic and can infect the patient, especially if the patient’s immune system is weakened, is on a catheter, or has an artificial limb/organ requiring the regular intake of immunosuppressants [58].

*S. saprophyticus* constituted 12.2% of the isolates; 13.6% of symptomatic women and 10.7% of asymptomatic women harboured *S. saprophyticus*. *S. saprophyticus* is commonly involved in urinary tract infections (UTIs); however, it is prevalent among sexually active young women [34], [59]. Little research has been conducted on the interaction of *S. saprophyticus* with the vagina, so data are scarce, unlike the information available on UTIs. One might suggest that the bacterium is capable of adhering to the vaginal epithelium and may contribute to the development of symptomatic infections of the genitourinary tract [60]. The exact way in which *S. saprophyticus* affects vaginal infections has not been fully elucidated, but it is believed to be due to its ability to withstand harsh and toxic environments, as well as its ability to adhere to vaginal epithelial cells through adhesins, surface proteins, and biofilm formation [61].

*S. aureus* was another common organism isolated in both apparently healthy women (3.8%) and those with vaginal infections (4.7%), with a tendentially higher prevalence observed in the latter group. This aligns with existing literature, which suggests that *S. aureus* can contribute to vaginal infections and may be a factor in symptom presentation in some cases [62], [63]. *S. aureus* is a potential pathogenic organism which can cause a variety of infections, particularly in the skin and soft tissues, across many different body sites [64], [65]. While *S. aureus* is recognized as a major pathogen in several infection types, its role in vaginal infections remains controversial [66]. This study demonstrates that *S. aureus* can reside in the vagina, and its presence may be considered a contributing factor to disease manifestation and pathogenicity [67], [68].

The overall incidence of *E. coli* (1.5%) was lower compared to previous research. For example, Dehkordi et al. [69] reported a 14.1% prevalence. Another study found a prevalence of 25% [70], while a cross-sectional study of sexually active Pakistani women using hormonal contraceptives estimated the prevalence at 20% [71]. In the current study, *E. coli* tended to be isolated more frequently in asymptomatic women (1.9%) compared to symptomatic women (1.2%). Most UTIs are caused by uropathogenic *E. coli*, accounting for nearly 80% of such infections [72], [73]. Given the anatomical proximity of the urinary and reproductive systems, infections can easily spread between the two. As previously noted, women with recurrent UTIs show higher levels of *E. coli* colonization in the vaginal area. This supports earlier findings that have linked UTIs with vaginal colonization of *E. coli* [69], [74]. The fact that *E. coli* was more frequently isolated from asymptomatic women compared to symptomatic women in this study raises intriguing considerations. Firstly, the presence of *E. coli* in the vaginal region of asymptomatic women may represent a transient or intermittent colonization rather than an active infection. Secondly, these individuals may have effective immune responses that prevent the development of symptomatic infections. The isolation of *E. coli* from high vaginal swabs, especially in asymptomatic women, highlights the importance of considering the vaginal microbiota as a potential reservoir for UTI-causing pathogens.

In the study, the overall prevalence of *Proteus* spp. was found to be 1.2%, while *K. pneumoniae* was isolated at a higher rate of 3.4%. *P. aeruginosa* was isolated at a rate of 2.7%. Specifically, *K. pneumoniae* showed a tendentially higher prevalence in women with symptoms (4.1%) of vaginal infections compared to those without symptoms (2.5%), while *P. aeruginosa* showed a tendentially higher prevalence in women with symptoms (3.6%) of vaginal infections compared to those without symptoms (1.9%). On the other hand, *Proteus* spp. were not detected in asymptomatic women. The higher prevalence recorded in symptomatic women indicate a potential association between the organisms and symptomatic vaginal infections.

The role of *Proteus* spp., *K. pneumoniae* and *Pseudomonas* spp. as etiologic agents of infections in humans extends beyond the urinary tract and includes various other clinical conditions [75], [76], while the focus of their involvement in vaginal infections is limited. These organisms are primarily known for their colonization in the lower human intestinal tract, with *Klebsiella* spp. being more prevalent [76]. Additionally, *Klebsiella* spp. can colonize the nasopharynx [77], [78]. The human digestive tract serves as a reservoir, leading to autoinfection or person-to-person transmission of nosocomial infections [78], [79]. Further research is necessary to fully understand the implications and prevalence of these organisms in vaginal infections.

*Sr. agalactiae* commonly known as Group B *Streptococcus* (GBS) is a bacterium known to be generally non-pathogenic and part of the normal microbiota in most asymptomatic adults. It is frequently isolated from the lower genital and gastrointestinal tract of asymptomatic women [80]. Nevertheless, under certain circumstances, it can act as an invasive pathogen and produce invasive infections in immunocompromised patients, for instance, in cases of bacteraemia [81]. In this study, *Sr. agalactiae* accounted for 4.9% of the isolates, indicating its presence among the study participants. It is noteworthy that the prevalence of *Sr. agalactiae* was slightly higher in symptomatic women (5.9%) compared to asymptomatic women (3.8%). This finding suggests a potential association between the presence of *Sr. agalactiae* and the manifestation of symptoms. *Sr. agalactiae* is also known to cause genital infections especially in pregnant women. It is believed that 10–30% of pregnant women are colonized with GBS and the bacterium can be isolated from the vagina or rectum. The colonization during pregnancy can become a danger to the foetus, since GBS can cross the birth canal and cause infections in a newborn, often leading to pneumonia, sepsis, and meningitis [82], [83]. While *Sr. agalactiae* is a normal human gastrointestinal and genitourinary flora, its ability to cause invasive infections in immunocompromised individuals and its role in maternal and neonatal health should not be overlooked. Effective screening, diagnosis, and management strategies are essential to mitigate the potential risks associated with *Sr. agalactiae* colonization and its potential to cause genital infections.

The overall prevalence of *Lactobacillus* spp. in the study was 8.23%. The prevalence was tendentially higher in asymptomatic group (9.4%) than in symptomatic group (7.1%). *Lactobacillus* spp. are well-known for their protective role in the vagina. A higher prevalence in the asymptomatic group (9.4%) suggests that a healthy population of *Lactobacillus* is associated with the absence of symptoms. This aligns with the established understanding that *Lactobacillus* help maintain vaginal health [1], [84], [85]. The lower prevalence of *Lactobacillus* in the symptomatic group could indicate a correlation between reduced *Lactobacillus* populations and the presence of symptoms, which may include vaginal discharge, itching, odour, or irritation [17]. However, the overall low prevalence of *Lactobacillus* spp. at 8.2% raises several important points about the factors influencing vaginal microbiota and the implications for women’s health. This finding contrasts with previous research, in which *Lactobacillus* was identified as the dominant species in the vaginal microbiota of healthy individuals even among Nigerian women [86], [87], [88]. Instead, this study observed a high prevalence of *E. faecalis* in the vaginal microbiota of the participants in both women with and without symptoms of vaginal infections. While *Lactobacillus* has been established as the most frequent organism in the vaginal environment, several studies from the Western hemisphere have revealed that the vaginal microbiota of African-American women have greater prevalence of anaerobic bacteria species compared to White and Caucasian women, and a decreased presence of *Lactobacillus* spp. [89], [90]. Black women are more likely to be defined as community-state type IV (CST IV) than any other ethnic group, and when a *Lactobacillus* spp. is found in an African American woman, it is likely to be *L. iners* [90]. Ravel et al. [51] characterized the vaginal microbiota of 396 asymptomatic North American women from four ethnic groups: Asian, Black, Hispanic, and Caucasian. They identified five distinct community-state types (CST I, II, III, IV, and V), most of which were dominated by *Lactobacillus* spp. CST I, II, III, and V were predominantly composed of* L. crispatus*, *L. gasseri*, *L. iners*, and *L. jensenii*, respectively, while CST IV was characterized by a diverse group of strict anaerobes [52].The low prevalence of *Lactobacillus* spp. in this study is concerning, yet it offers valuable insights in Nigeria. The high prevalence of *E. faecalis* suggests the need for further research into its role in vaginal health, including its potential probiotic properties. The significant difference (*p=0.045*) in *P. mirabilis* frequency between asymptomatic and symptomatic groups suggests that this organism is more likely to be associated with symptomatic cases. For organisms such as *E. faecalis*, *S. epidermidis*, and *S. saprophyticus*, the lack of significant differences (p>0.05) suggests these bacteria may colonize both symptomatic and asymptomatic individuals without a clear link to symptom manifestation. These organisms could be part of the normal microbiota or opportunistic pathogens whose presence does not always correlate with clinical symptoms. For example, *E. faecalis* and *S. epidermidis* are common commensals that may only cause infection under certain conditions (e.g., immunosuppression, catheter use) [56]. *S. saprophyticus* is known to cause UTIs but might also exist harmlessly in some individuals. The non-significance differences in the prevalence of *S. agalactiae* or *K. pneumonia* between asymptomatic and symptomatic groups, might be due to insufficient sample size or variability in the groups. Larger sample sizes might reveal more subtle differences that were not detectable in this analysis. This study of the vaginal microbiota of women attending the Jos University Teaching Hospital emphasizes the importance of investigating the implications of *Lactobacillus* deficiency for women’s health and highlights the need to consider diverse populations in order to gain a comprehensive understanding of vaginal microbiota composition and its impact on women’s health globally.

### Limitations

First, the diagnosis of BV relied on the Nugent score, which can introduce subjectivity and inter-observer variability, potentially affecting the consistency of BV prevalence rates reported. 

Second, the use of a culture-based approach to assess bacterial diversity may not capture the full spectrum of vaginal microbiota, particularly non-cultivable or fastidious bacteria, leading to an incomplete understanding of the microbial landscape.

Additionally, the sample size (220) might not adequately represent diverse sociodemographic groups in the broader community, and the cross-sectional design restricts causal inferences about the relationships between bacterial species and BV, providing only a snapshot of the bacterial diversity rather than a comprehensive view of its dynamics over time.

Furthermore, the lack of advanced molecular techniques, such as 16S rRNA sequencing, restricts the identification of a broader range of bacterial species, possibly overlooking significant taxa that contribute to vaginal health. Lastly, the study did not comprehensively assess potential confounding variables, such as hormonal status and immune responses, limiting the understanding of the various determinants influencing women’s vaginal health.

## Conclusion

While the study found no associations between sociodemographic factors, lifestyle, sexual practices, or hygiene practices and the prevalence of BV, it highlights the multifactorial nature of the condition. The observed diversity in vaginal microbiota, particularly the higher prevalence of *E. faecalis* in asymptomatic women, suggests the complexity of microbial interactions. The worryingly low prevalence of *Lactobacillus* spp. indicates a potential risk for infections, while the presence of potentially pathogenic bacteria, e.g., *S. saprophyticus* and *S. agalactiae*, underscores the need for further research. Overall, understanding these dynamics is crucial for developing effective healthcare interventions for managing BV.

## Notes

### Authors’ ORCIDs

- Florence Yachim Danjuma: 0009-0009-5668-1419
- Michael Macvren Dashen: 0009-0007-9587-0314
- Anayochukwu Chibuike Ngene: 0000-0003-4730-2834
- Otumala John Egbere: 0009-0001-6908-763X

### Ethical approval 

The study received ethical approval from the JUTH ethics committee (Ref. JUTH/DCS/IREC/127/XXX/2478). Oral informed consent was obtained from the participants before enrollment. All the procedures conducted complied with the ethical standards of the Federal Ministry of Health in Nigeria.

### Funding

None. 

### Competing interests

The authors declare that they have no competing interests.

## Figures and Tables

**Table 1:**
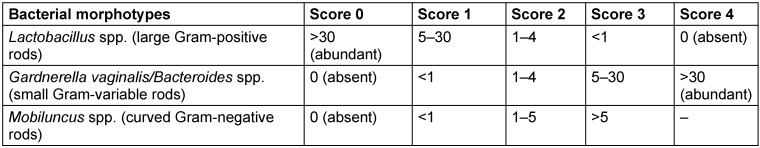
Scoring of bacterial morphotype on Gram-stained smear

**Table 2:**
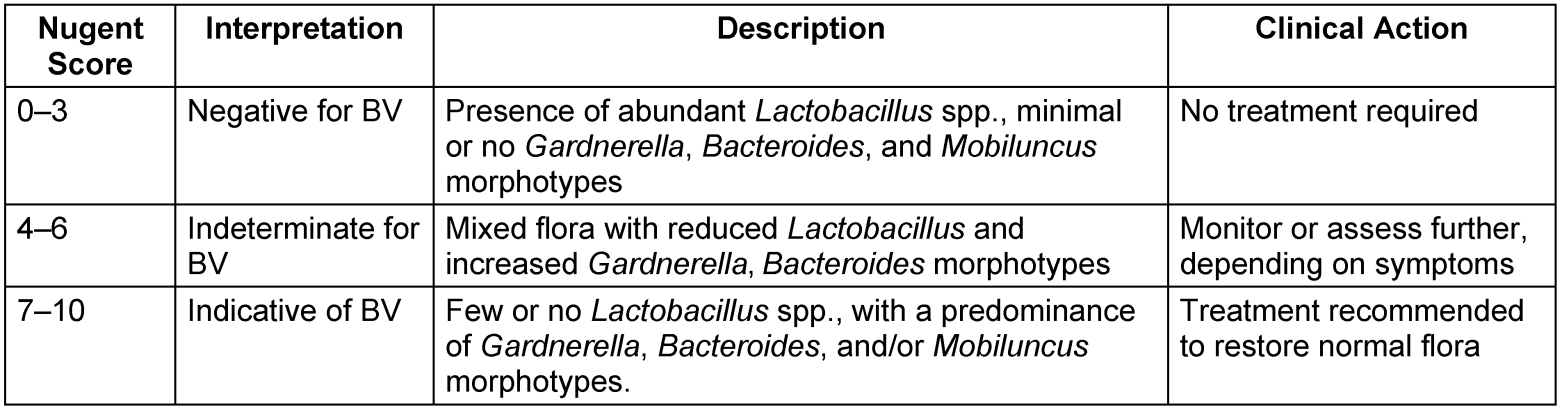
Interpretation of Nugent score

**Table 3:**
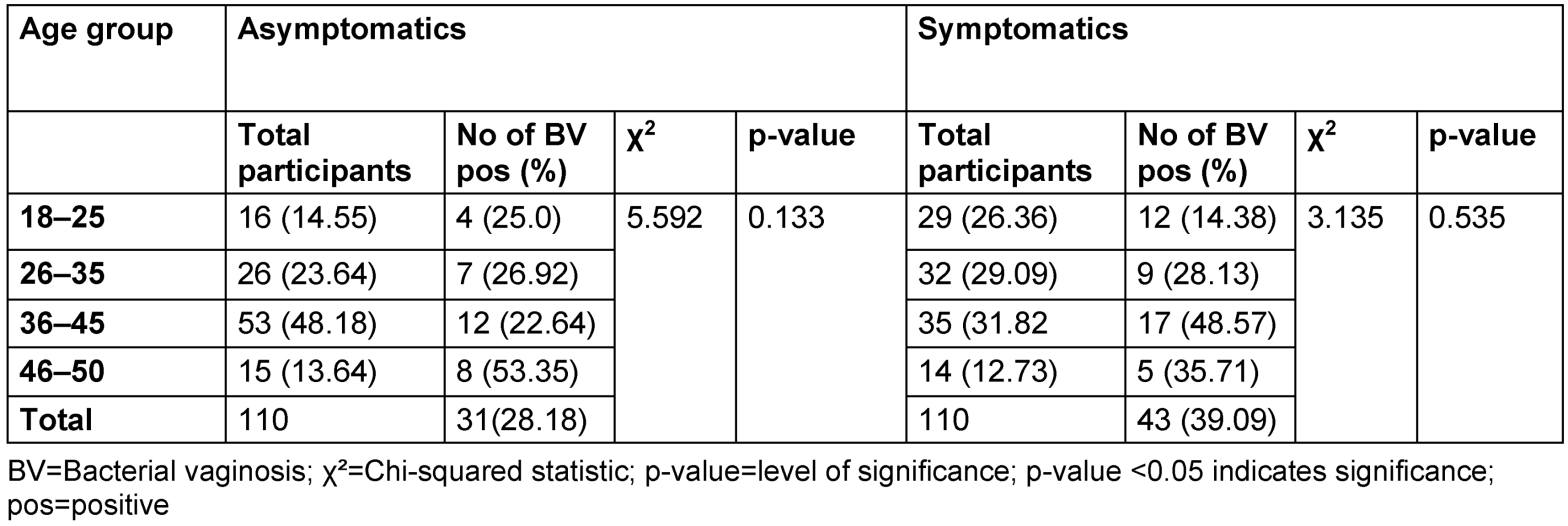
Prevalence of bacterial vaginosis by age

**Table 4:**
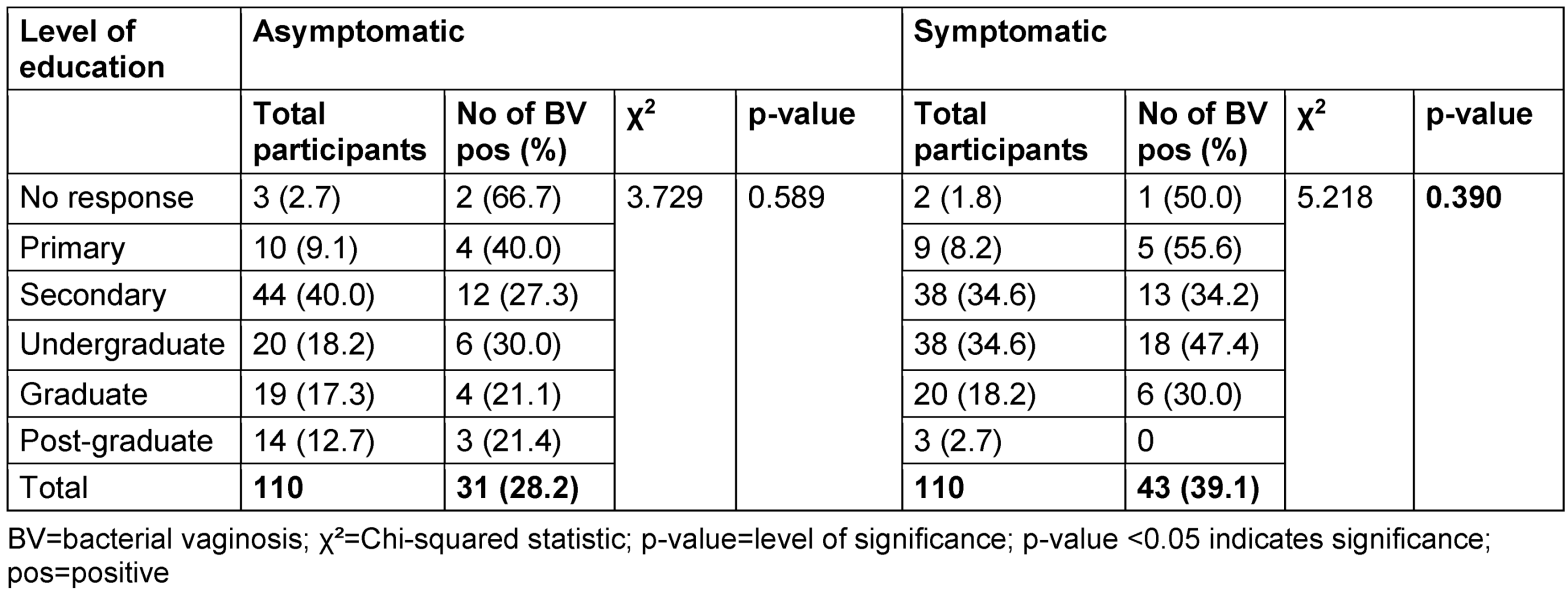
Prevalence of bacterial vaginosis by level of education

**Table 5:**
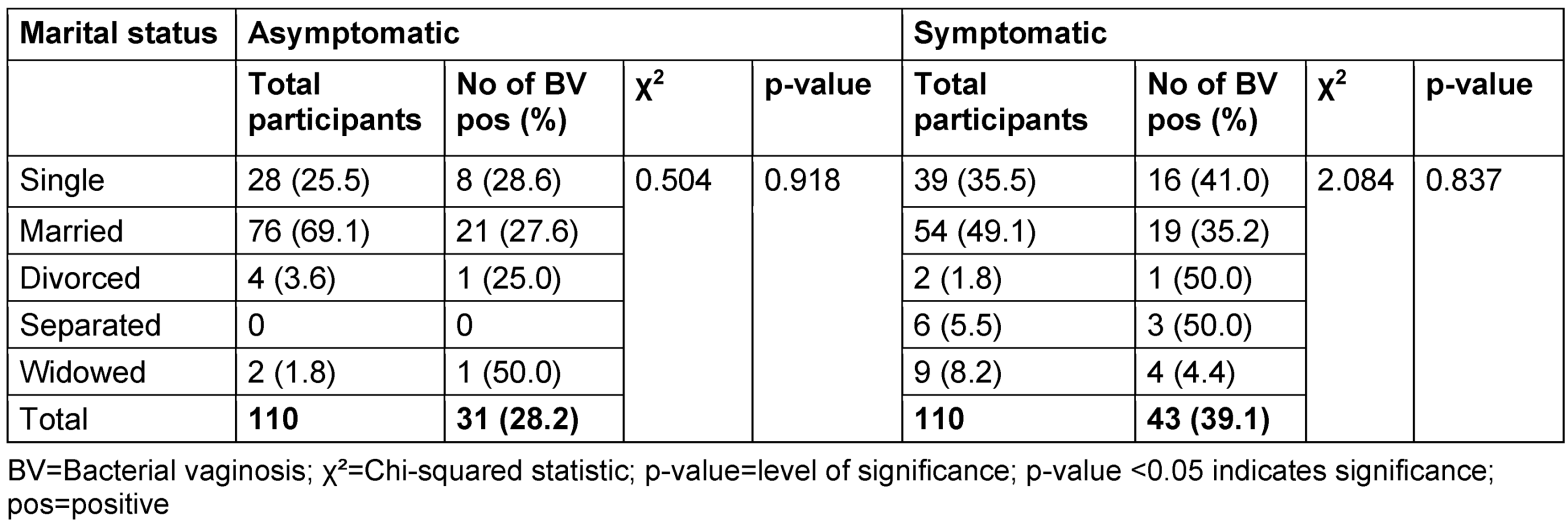
Prevalence of bacterial vaginosis by marital status

**Table 6:**
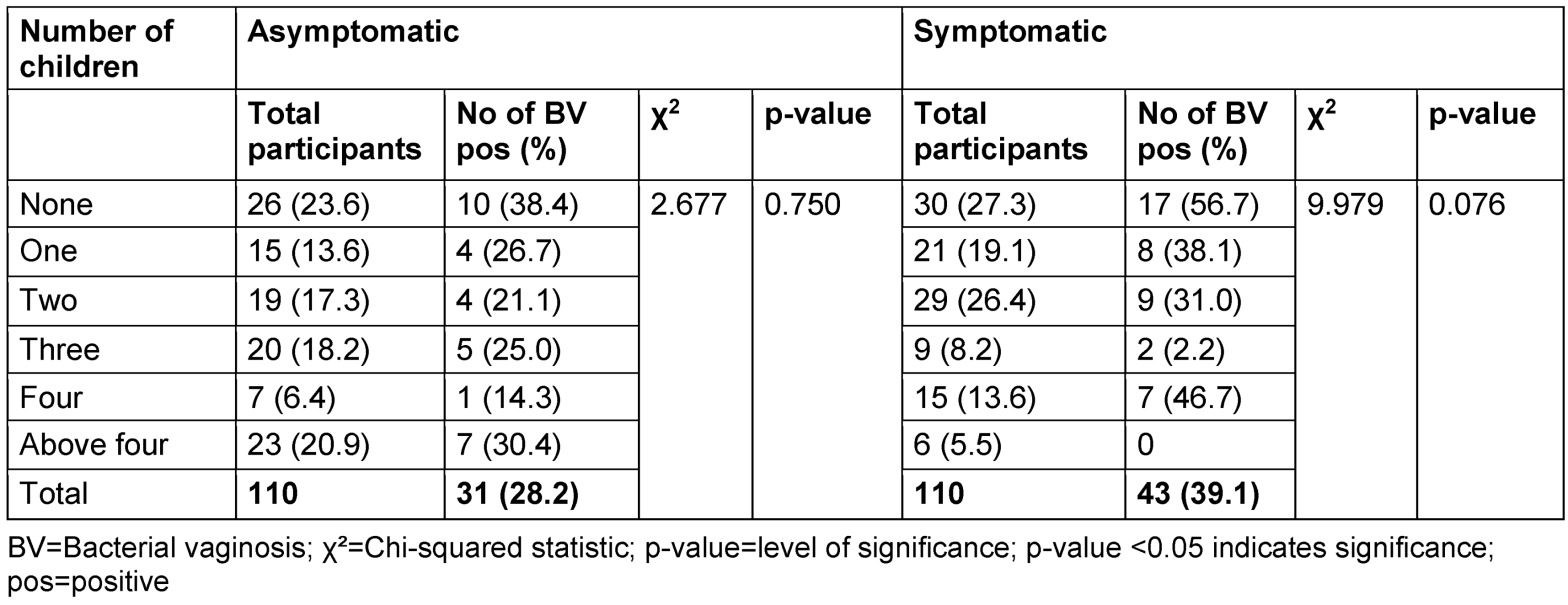
Prevalence of bacterial vaginosis by number of children

**Table 7:**
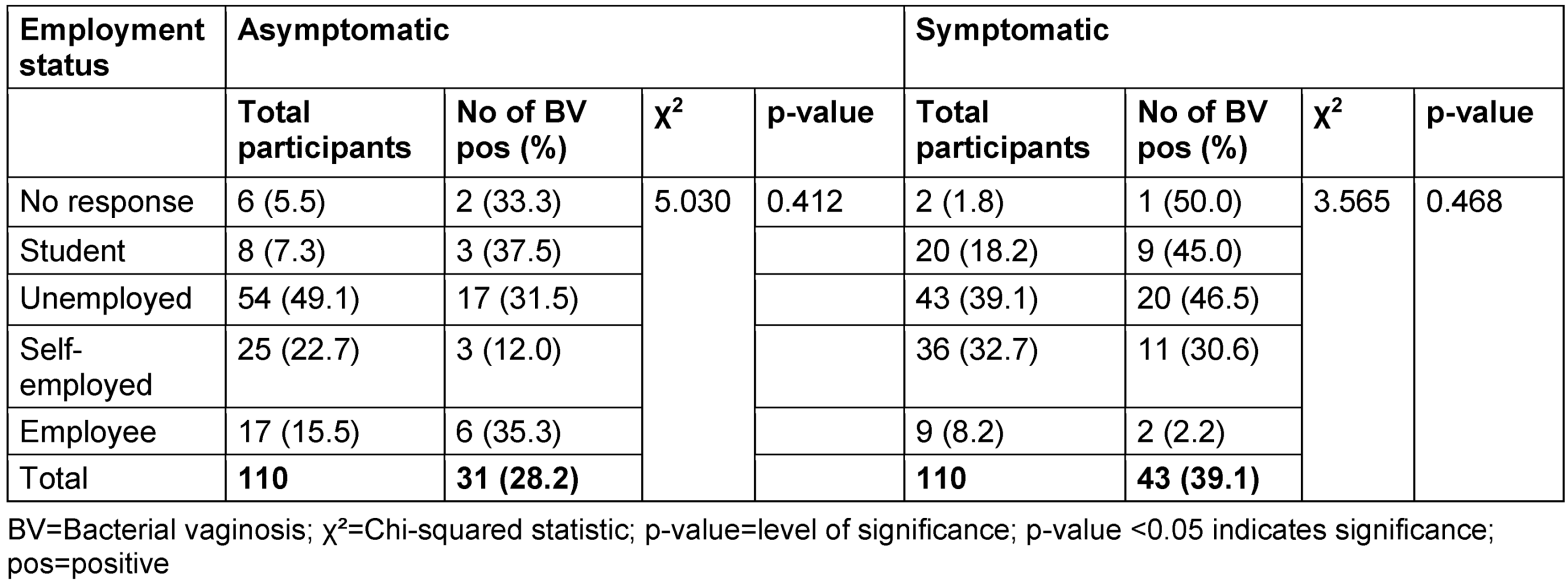
Prevalence of bacterial vaginosis by employment status

**Table 8:**
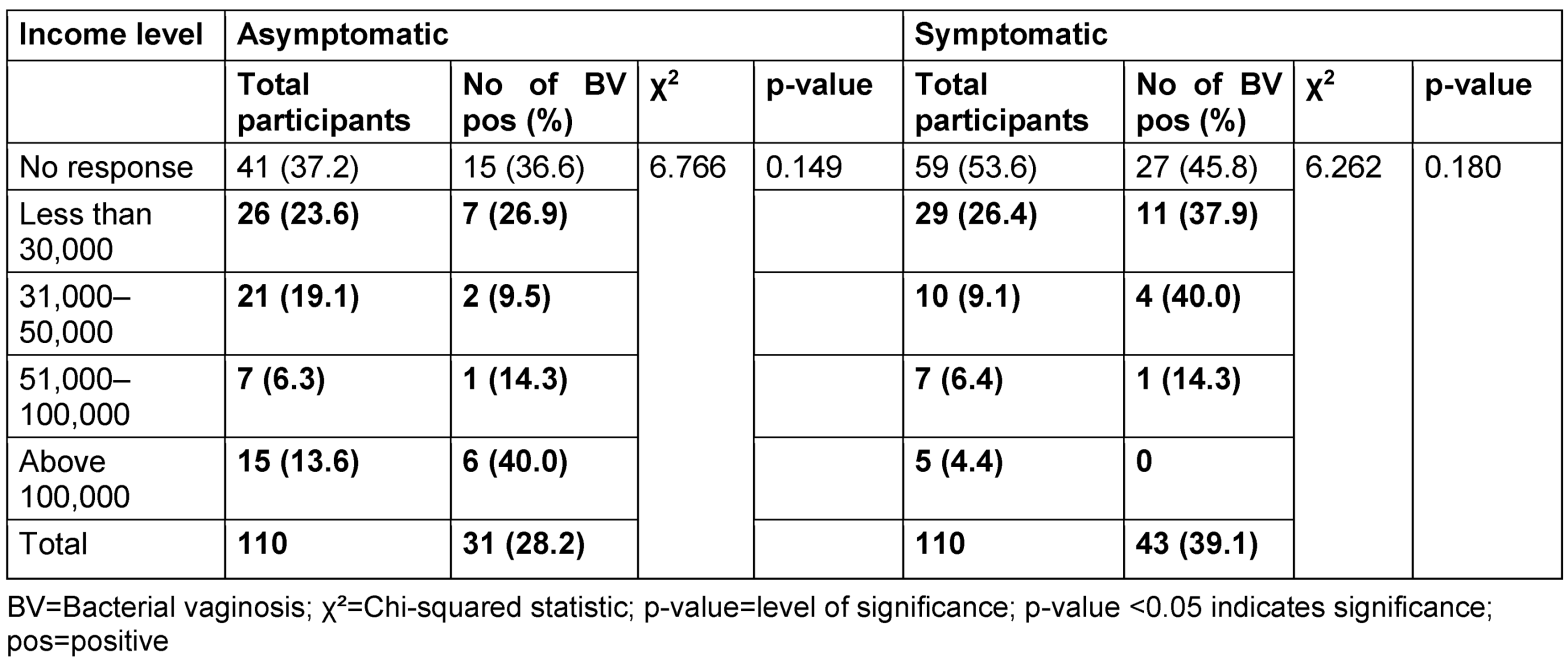
Prevalence of bacterial vaginosis by income level

**Table 9:**
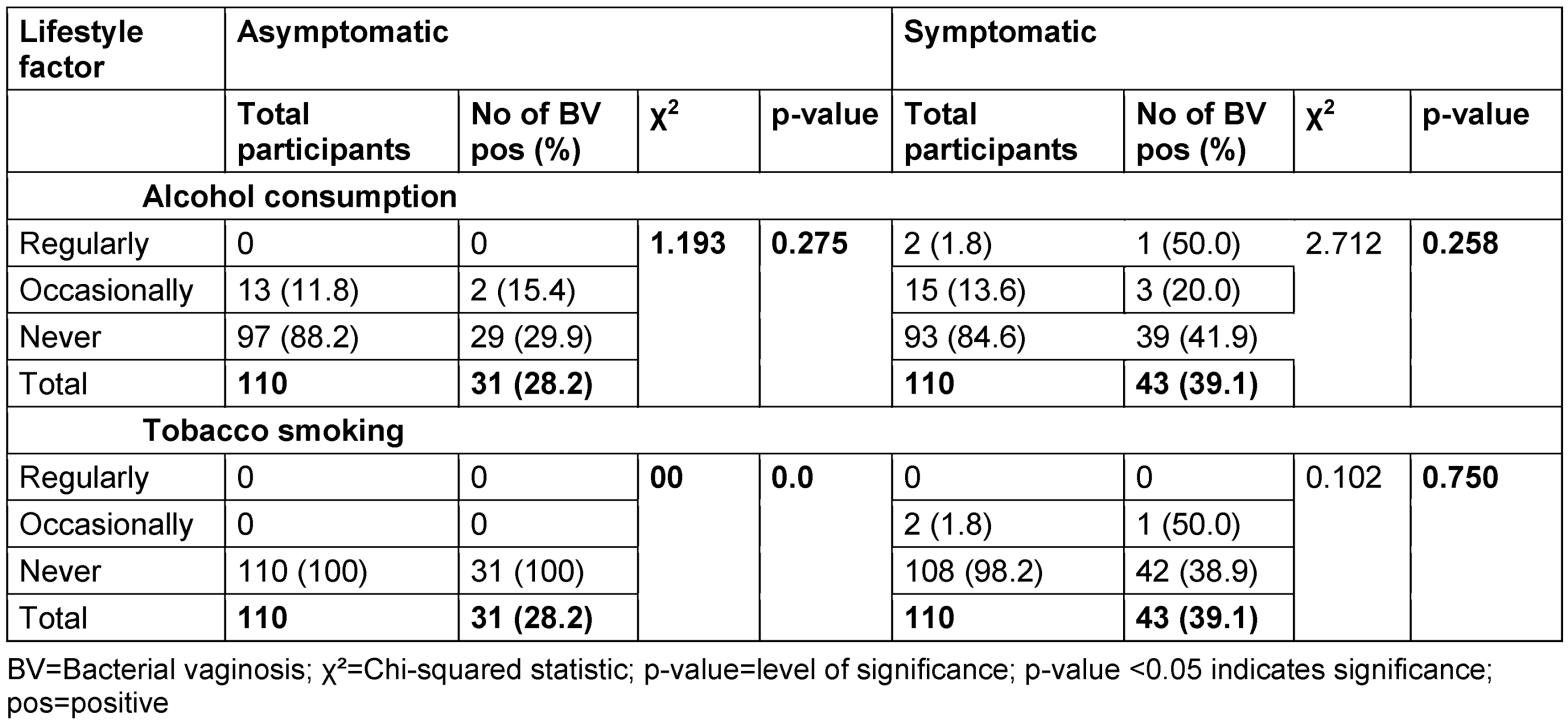
Prevalence of bacterial vaginosis by lifestyle factors

**Table 10:**
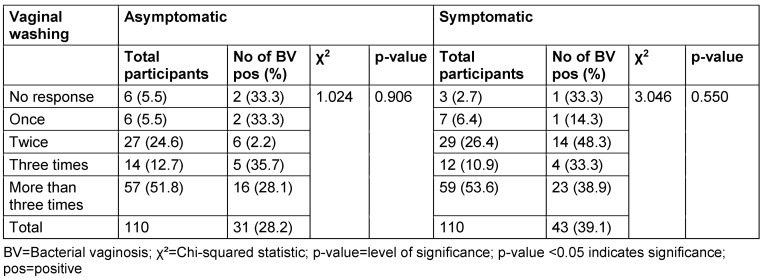
Prevalence of bacterial vaginosis by frequency of vaginal washing

**Table 11:**
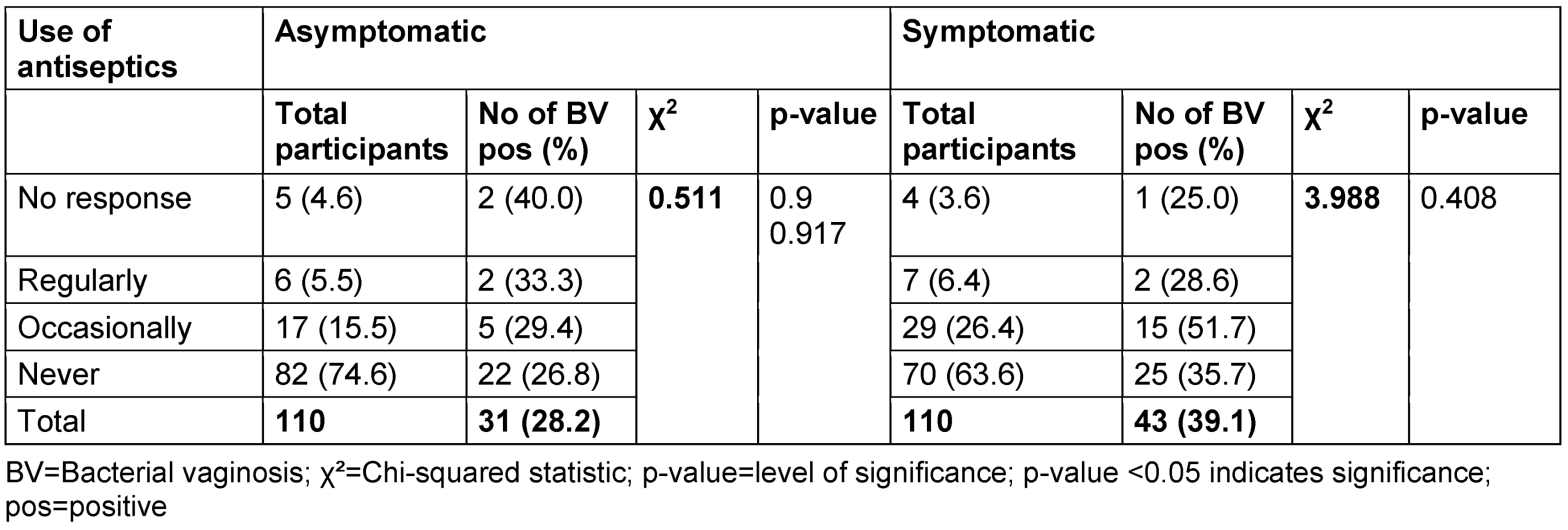
Prevalence of bacterial vaginosis by use of antiseptics

**Table 12:**
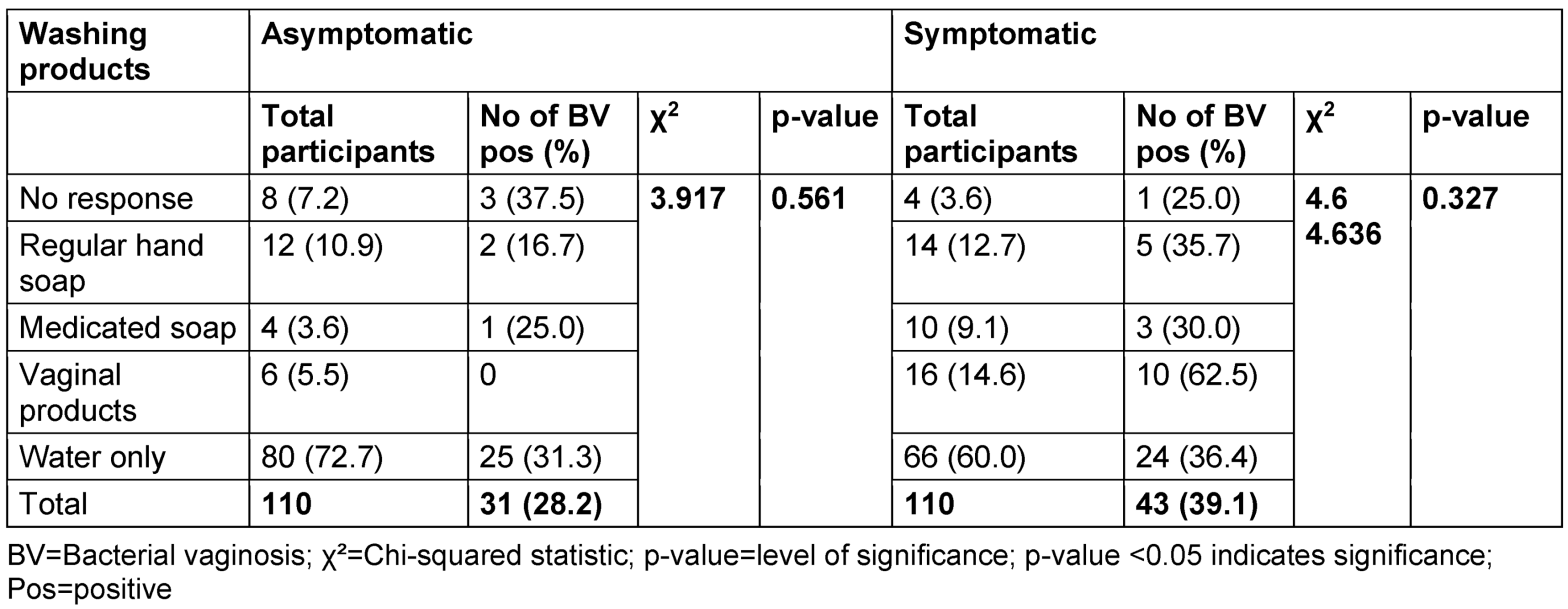
Prevalence of bacterial vaginosis by specific products used for vaginal washing

**Table 13:**
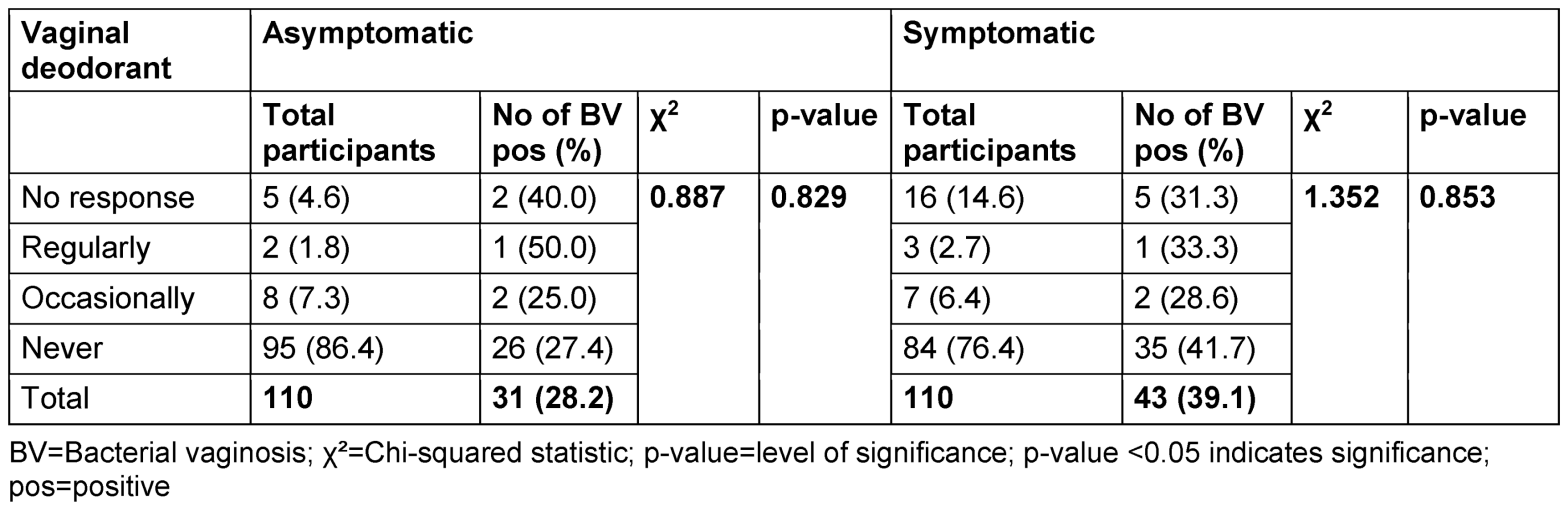
Prevalence of bacterial vaginosis by use of vaginal deodorant

**Table 14:**
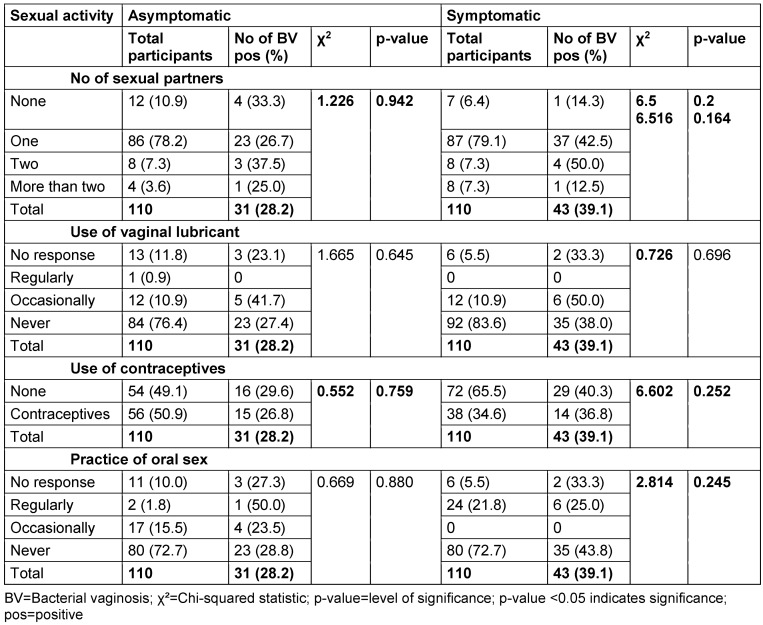
Relationship between sexual activities and bacterial vaginosis

**Table 15:**
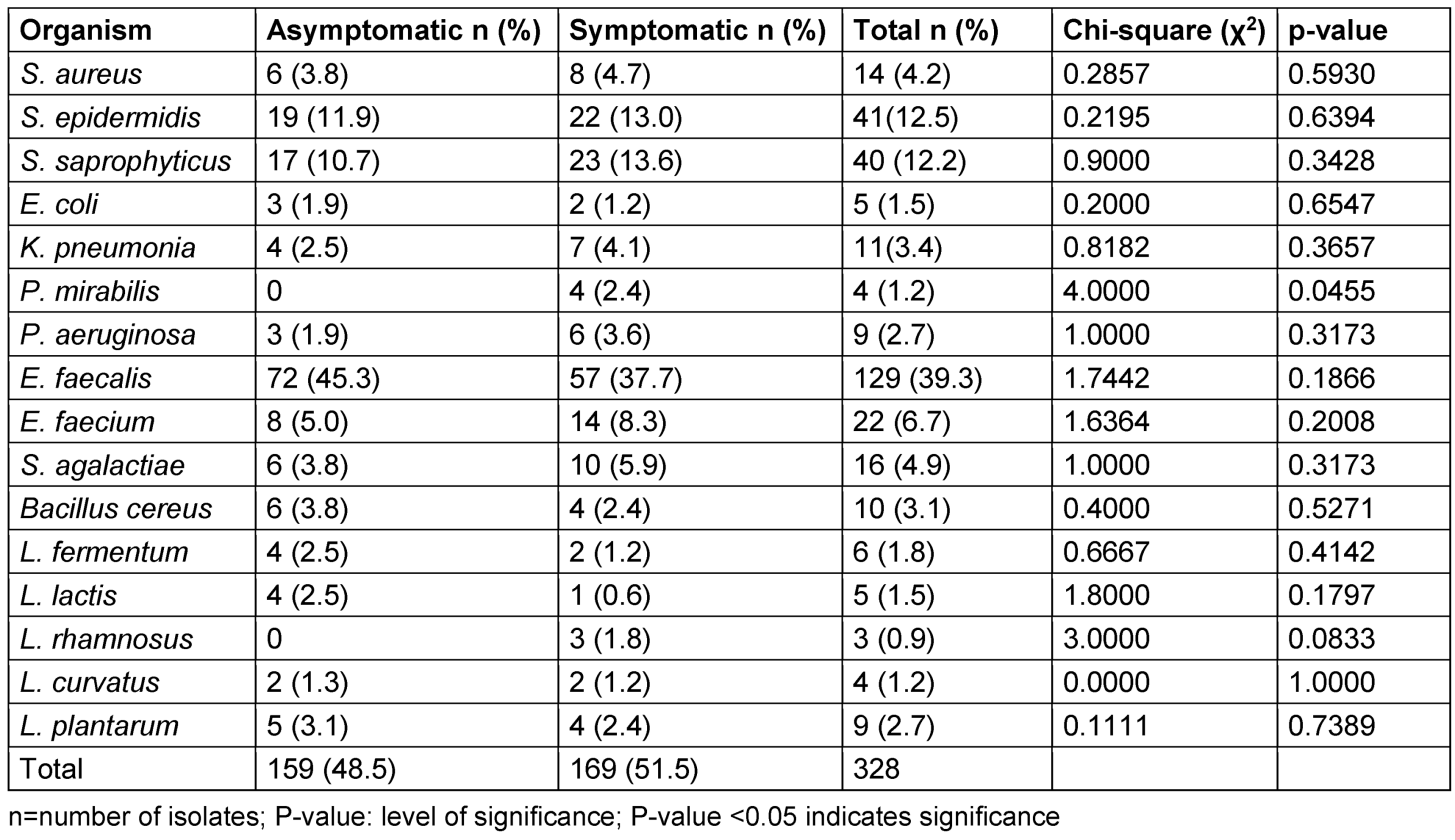
Frequency of occurrence of bacterial species isolated in the asymptomatic and symptomatic groups
